# Unmet Needs in the Management of Cervical Dystonia

**DOI:** 10.3389/fneur.2016.00165

**Published:** 2016-09-28

**Authors:** Maria Fiorella Contarino, Marenka Smit, Joost van den Dool, Jens Volkmann, Marina A. J. Tijssen

**Affiliations:** ^1^Department of Neurology, Leiden University Medical Centre, Leiden, Netherlands; ^2^Department of Neurology, Haga Teaching Hospital, The Hague, Netherlands; ^3^Department of Neurology, University Medical Center Groningen, University of Groningen, Groningen, Netherlands; ^4^Faculty of Health, ACHIEVE Centre of Applied Research, Amsterdam University of Applied Sciences, Amsterdam, Netherlands; ^5^Department of Neurology, Academic Medical Center, Amsterdam, Netherlands; ^6^Department of Neurology, University Clinic of Würzburg, Würzburg, Germany

**Keywords:** cervical dystonia, botulinum toxin, deep brain stimulation, physical therapy modalities, non-motor features

## Abstract

Cervical dystonia (CD) is a movement disorder which affects daily living of many patients. In clinical practice, several unmet treatment needs remain open. This article focuses on the four main aspects of treatment. We describe existing and emerging treatment approaches for CD, including botulinum toxin injections, surgical therapy, management of non-motor symptoms, and rehabilitation strategies. The unsolved issues regarding each of these treatments are identified and discussed, and possible future approaches and research lines are proposed.

## Introduction

Cervical dystonia (CD) is the most prevalent form of adult-onset focal dystonia, and is characterized by abnormal postures of head and neck, that can considerably impair daily living.

There are several unmet needs in the management of CD. In this article, we focused on four main aspects of the treatment of this disorder, including botulinum toxin injections, surgical therapy, management of non-motor symptoms (NMS), and rehabilitation strategies.

For each of these issues the state-of-the art is presented and some of the current knowledge gaps are highlighted. In addition, we propose potential research lines that could be developed to manage these issues.

## Botulinum Toxin

### What Is Known?

Botulinum neurotoxin (BoNT) injections are the treatment of choice for CD.

There is class I evidence to support efficacy and safety of the three commercially available formulations of BoNT-A (onabotulinumtoxinA, abobotulinumtoxinA, and incobotulinumtoxinA) (1–3), and of BoNT-B (rimabotulinumtoxin B) (4).

As much as 70–85% of the patients report a significant benefit from the treatment (5). Efficacy on motor symptoms varies from 20 to 70%, based on the assessing method used. Significant improvement is also documented on pain and quality of life (QoL) (6).

Although BoNT treatment is routinely performed worldwide and is satisfying for many patients, the obtained effect is still far from optimal. In addition, BoNT treatment is in some cases associated with the occurrence of side effects, such as dysphagia or excessive muscle weakness. These side effects are due to an excessive dose of BoNT or to the spread of BoNT to adjacent structures, and may limit the efficacy of the treatment.

### What Is Uncertain?

In order to further improve the efficacy and safety of the treatment, the accurate placement of the minimum effective dose of toxin in the dystonic muscles should be ensured. At present, there is still no agreement on a recommended starting dose or on the minimum effective dose per muscle.

Moreover, there is still great variability concerning treatment strategies. Multi-point BoNT injections have been proposed as more effective than single point injections (7), but convincing evidence on these topics is still lacking.

The use of polymyography to identify dystonic muscles before treatment, and the use of electromyography (EMG) to guide injections, has been proposed to improve the accuracy of BoNT delivery. While some studies show that this approach may provide a significant advantage in BoNT-naïve patients (8, 9), as well as in patients unsatisfactorily treated with standard injections (10, 11), this still need to be further confirmed in larger series. Moreover, the modalities and indications of the neurophysiological approach need to be further specified.

The use of imaging techniques has also been proposed to identify the dystonic muscles before treatment and to improve the accuracy of the placement of BoNT. Preliminary reports suggest that the use of ultrasound-guided injections might help localizing the target muscles and reducing the episodes of dysphagia in patients who had experienced it with standard treatment (12).

A number of patients do not respond to BoNT treatment, or develop a secondary resistance. A currently accepted definition of secondary non-responsiveness implies “insufficiently improved posture after three or more unsuccessful injection cycles in CD patient’s previously achieving satisfactory results” (13).

Change in CD pattern across time, with the appearance of more complex multiaxial dystonic movements or tremor, account for some of the non-responders. Another well-known cause of non-responsiveness is the development of antibodies against BoNT formulations (14). This issue has been described with different BoNT formulations, including onabotulinumtoxinA, abobotulinumtoxinA, and rimabotulinumtoxinB (15), while it does not seem to be a concern when incobotulinumtoxinA is used (16). At present, there is no agreement on the strategies to avoid the formation of antibodies. Although this problem likely occurs only sporadically, a minimum safe interval of 12 weeks or longer is still used in most centers (17). This strategy, however, limits treatment of a larger number of patients, who report reemergence of symptoms before this time. The safety of shorter intervals between injections and of the so-called booster injections still needs to be explored.

Another unsolved and largely debated practical issue concerns the optimal conversion ratio between different formulation of BoNT-A, or between BoNT-A and BoNT-B.

Based on studies using different methodology, a conversion of onabotulinumtoxinA to abobotulinumtoxinA 1:3 IU (18, 19), as well as ratios of 1:2.5 (20) have been proposed over time, while a conversion ratio of 1:1 is proposed for onabotulinumtoxinA to incobotulinumtoxinA.

### Future Perspectives

Future research lines should focus on improving the benefit/side effects ratio of BoNT treatment and on reducing the rate of primary and secondary non-responsive patients.

A standardized working definition of non-responsiveness should be developed, which should take into account an objective measure of the lack of improvement as well as an evaluation of the appropriateness of BoNT treatment. An objective and universally accepted working definition would be of crucial importance to assess new treatment strategies and to identify patients for whom more invasive (surgical) treatment are indicated.

Dose-finding studies and comparative studies across different toxins should be performed. The additional value of neurophysiology and imaging in improving the intramuscular placing of BoNT should be explored. In order to minimize patients’ discomfort, the minimum safe interval between treatments should be determined.

## Surgical Treatment

### What Is Known?

Deep brain stimulation (DBS) of the internal globus pallidus (GPi) is an established surgical treatment for patients with generalized dystonia (21, 22). Because the initial studies suggested an equally beneficial effect for all body regions, the method was soon applied to patients with focal or segmental dystonias, who no longer responded to BoNT.

Krauss was the first to describe the beneficial outcome in three patients with CD in 1999 (23). Meanwhile three controlled clinical studies were conducted evaluating GPi-DBS in CD patients who failed on medical treatment: a Canadian prospective, multicenter and observer-blinded study assessed 10 CD patients who were further followed for 12 months (24). Motor improvement was 28% at 6 months and 43% at 12 months (TWSTRS motor score). Pain and disability scores were also improved by 66 and 64%, as well as mood [Beck’s Depression Inventory (BDI)] and QoL (SF-36) by 58 and 24%, respectively. Another prospective single-center study followed eight CD patients for up to 48 months after GPi-DBS (25), reporting a median reduction in the TWSTRS motor score of 50% at 6 months and of 73% at last follow-up. The only randomized sham-controlled multicenter study of bilateral GPi-DBS in CD followed patients for a total of 6–9 months after surgery (26). Sixty-two patients were implanted with a neurostimulation system and randomly assigned to either active or sham stimulation (stimulator output 0V). After 3 months, TWSTRS severity score was reduced by 26% in the treatment group compared to 6% in the sham group. There was a 3.8 point difference between both groups, which was significant. TWSTRS disability score and Bain tremor score were also significantly improved in the neurostimulation group, whereas TWSTRS pain score and QoL (Craniocervical Dystonia Questionnaire-24 score) were not different. Evaluations were repeated in all patients after receiving 6 months of effective neurostimulation. At the follow-up, significant improvements compared to the pre-surgical baseline were found for TWSTRS severity score (28%), disability score (46%) and pain (51%), Tsui score (57%), Bain tremor score (66%), and global dystonia ratings by patients (49%) or physicians (53%). BDI was reduced by 20%, the cranio-cervical dystonia questionnaire-24 showed a 28% improvement. No permanent adverse effects were found. Transient adverse effects included device infection (*n* = 3), misplacement/dislocation of electrodes (*n* = 3) or neurostimulator (*n* = 1), stroke/hemorrhage (*n* = 1), and seizure (*n* = 1). Four patients claimed pain at the extension cable. The most frequent stimulation-induced side-effect was dysarthria (seven patients). Stimulation-induced bradykinesia was observed in one patient, but has previously been described as a relevant adverse effect of pallidal neurostimulation in several series (27, 28).

It has been suggested that the subthalamic nucleus could be a better target for DBS in CD with equal motor benefit but less risk of stimulation-induced parkinsonism (29).

### What Is Uncertain?

Larger series are needed to ascertain which types of CD respond best to pallidal DBS, and to assess predisposing factors and the true prevalence and risk factors of stimulation-induced parkinsonism. Subthalamic stimulation, which was forwarded as an alternative, induces (transient) dyskinesia in a large proportion of patients and the cognitive and behavioral safety has not been evaluated yet. So far, DBS has been advocated only in patients no longer responding to BoNT treatment, as a last line therapy. A comparative trial of BoNT treatment in comparison to DBS has not been performed yet.

### Future Perspectives

Registry data of DBS surgery in CD would help to evaluate outcomes in daily practice, define responder profiles, and assess the frequency of less common adverse effects. The effect of DBS on non-motor features should be systematically assessed. Randomized controlled trials (RCTs) are needed to compare pallidal and subthalamic neurostimulation and DBS in general vs. best conservative management of CD.

## Management of Non-Motor Symptoms

### What Is Known?

Growing evidence suggests that the phenotype of dystonia includes also NMS, which could in part account for the reduced QoL in CD (30, 31).

Sensory abnormalities are the most frequently NMS associated with CD. The onset of motor symptoms can be preceded by a feeling of discomfort in the neck and dystonic movements are sometimes interpreted as an attempt to decrease this feeling (32). Involvement of the sensory system is also indicated by the *geste antagoniste*, which modifies cortical EEG activity and GPi local field potentials, even before touching the head (33). Furthermore, several studies found abnormalities in temporal and spatial discrimination thresholds in CD patients, both in affected and unaffected body parts, and in unaffected first-degree relatives (34, 35).

Pain is present in up to 90% of CD patients, which is rated as moderate to severe by 70% (36). Two-third of the patients use analgesics. Pain might be a consequence of motor symptom severity (37), but could also be influenced by depressive and anxiety symptoms (31). It is proven that BoNT treatment as well as surgical treatments, such as DBS (26) or selective peripheral denervation (38), significantly improves pain associated with CD (36, 37).

The prevalence of psychiatric disorders in CD can reach up to 91.4%, compared to 35% in the general population (39). This could logically be the consequence of living with a chronic, visible, and invalidating disorder. However, compared to the prevalence of psychiatric symptoms in other chronic and visible diseases, such as alopecia areata, CD patients still have a significantly increased odds ratio to develop psychiatric co-morbidity (40). The most prevalent psychiatric disorders include depressive symptoms (40–45), anxiety symptoms/panic disorders (39, 40, 44, 45), obsessive–compulsive symptoms (41, 45) and substance abuse (45). Importantly, a few studies showed that psychiatric co-morbidity is the most important predictor of poorer health-related QoL, especially for the domains general health, role functioning, bodily pain, and emotional and mental health (31, 46, 47).

At this moment, no treatment trials have been described with the aim to directly improve psychiatric symptoms in CD patients.

### What Is Uncertain?

The prevalence and characteristics of the different NMS in CD, including sleep disturbances and cognition, have not been systematically studied and existing studies show contrasting results. A recurring debate is whether NMS are a direct consequence of the motor symptoms of dystonia or intrinsic to the neurobiology and thereby part of the phenotype.

Cervical dystonia patients showed an impaired sleep quality compared to healthy controls: in two studies, this was correlated with depressive symptom scores (48, 49), while in one study it appeared to be independent from psychiatric disorders and medication use (50). Successful BoNT treatment did not improve sleep quality, arguing against a secondary discomfort due to the dystonia motor symptoms (50). Excessive daytime sleepiness was detected in one study, but at least in part explained by the use of anticholinergic drugs (51). Other studies did not find significant differences in daytime sleepiness (48, 49).

Studies concerning cognitive impairment in CD are still very limited. One study showed impairments in the domains working memory, processing speed, visual motor ability, and short-term memory (52). Other small studies found impairment of visuospatial function (53) and a sustained attention deficit, the latter disappearing after BoNT treatment (54).

Convincing data support a disruption of sensory-motor system also in healthy first-degree relatives of dystonic patients, suggesting a possible endophenotype (55). For example, temporal discrimination threshold (TDT) was found abnormal not only in about 80% of dystonia patients but also in about 50% of first-degree female relatives older than 48. In male relatives, the penetrance was reduced (34, 56).

The onset of psychiatric disorders before the onset of the movement disorder in ~70% of the cases (42, 44, 45) is one of the strongest arguments toward a shared pathophysiology. This is also supported by a men-to-women ratio of psychiatric disorders of 1:1 in CD patients compared to 1:2 in the general population, higher incidence of psychiatric disorders in CD patients compared to other visible and chronic disorders, and different personality profiles found in CD patients, which develop long before adolescence and onset of motor symptoms (35).

Drawing firm conclusions on the etiology of NMS in CD remains difficult, also considering the tight correlation between pain, psychiatric symptoms, sleep disturbances, and motor symptoms.

### Future Perspectives

In order to solve the issue of the etiology of NMS in CD, prospective studies are necessary. Selecting an appropriate group for prospective studies has proven challenging. This might change with the identification of genetic forms of CD, such as the *GNAL* and *ANO3* gene (57–60), which would allow studying homogeneous clinical subgroups, even in the pre-symptomatic phase.

Another strategy could be the identification of endophenotypes in larger groups, based on biomarker, such as the TDT.

Clinical trials are required toward the effect of treatment of NMS on health-related QoL.

## Rehabilitation Strategies

### What Is Known?

Evidence toward the effectiveness of rehabilitation strategies is scarce. Two systematic reviews described the effects of different rehabilitation strategies in various forms of primary dystonia (61) and CD alone (62), suggesting that multimodal physical therapy (PT) programs, added to BoNT treatment, further improve disability and pain compared to BoNT treatment alone (61, 62). Only three clinical trials (63–65) and one case–control study (66) investigated the effects of a multimodal PT program in combination with BoNT treatment.

One single-blind RCT in 40 patients showed significant improvements on pain and daily-life activities, and a prolonged duration of the BoNT effect, after a 6-week PT program of active exercises, muscle stretching and massage compared to BoNT treatment alone (63). A second single-blind RCT in 40 patients showed decreased disability and a significant decrease of head deviation and improved hand functions after a 6-week PT program of active exercise, muscle stretching, and TENS in addition to BoNT treatment (64). The third single-blind RCT of 20 patients found only a trend toward greater improvement on head posture, pain, and disability in the group that received 12 weeks of active exercise, relaxation, and BoNT treatment compared to the group that received relaxation and BoNT treatment only (65).

One case–control study followed 40 patients in a 4-week PT program of active exercise, muscle stretching, active and passive neck mobilizations, and electrostimulation of the dystonic muscles in adjunction to BoNT treatment, or BoNT treatment alone. The PT group showed significantly more improvement on pain, and on some subscales of the SF-36 (66).

### What Is Uncertain?

The available results should be interpreted with caution. The content of PT programs varied across studies, including motor learning exercises [Bleton method (67)], passive or active mobilization techniques of the cervical spine, stretching of the dystonic muscles, relaxation, and electrotherapy, such as EMG biofeedback or TENS. It is, therefore, difficult to identify the most effective intervention or combination of interventions.

Frequency and duration of PT sessions also varied from 40 min every other day for 6 weeks (64), 75 min 5 days a week for 5 weeks (66), 90 min a day for 2 weeks (63) up to a 12-week program with a weekly 30-min session during the first 4-weeks, and a session every fortnight for the remaining 8 weeks (65). Besides, current studies mainly show short-term effects associated with brief and intensive PT programs (63, 64, 66), which could be difficult to implement in current regular care of a chronic disease, such as CD. The long-term effects of less intense and longer PT programs have not been explored yet.

### Future Perspectives

Future research should focus on standardized PT programs that are effective but also adequate to treat patients with a chronic conditions and an active life. PT programs with longer treatment periods and the emphasis on self-management of symptoms and the ability of patients to improve their performance of daily life tasks should be the focus. Currently, such a PT program is being investigated in a large Dutch RCT (68).

The effect of PT interventions on the pathophysiological mechanisms of CD should also be studied. Although the pathophysiology of CD remains largely unclear, maladaptive neuroplastic changes may play an important role (69). By integrating PT programs with modern training principles that have proven relevant for neural rehabilitation and motor learning, these deficit may be altered (70–74).

Additionally, high-quality research combining electrophysiological parameters or imaging techniques with clinical outcomes can help to further unravel the effects of PT programs on CD.

## Final Considerations

There are still many unmet needs in the management of CD. A better understanding of the pathophysiology of CD is necessary to plan new treatment strategies and to improve existing treatments. In addition, the available rating scales for CD have some clinimetric issues and do not equally address all the domains of the disease. This points to a need for updated scoring instruments in order to support studies on the pathogenesis and progression of the disease and to more accurately evaluate the outcomes of clinical trials. Specific standardized rating scale for NMS in (cervical) dystonia should also be developed.

Finally, it is widely accepted that motor improvement is not the only determinant of treatment success in CD: pain, social distress, and psychological factors play sometimes a greater role toward patient satisfaction. This calls for a multi-disciplinary approach posing more attention to the subjective determinants of QoL in CD.

## Author Contributions

All the authors (MC, MS, JD, JV, and MT) provided substantial contributions to the conception or design of the work; drafted part of the manuscript and revised the rest of the manuscript critically for important intellectual content; approved the final version to be published; agreed to be accountable for all aspects of the work.

## Conflict of Interest Statement

MC, Advisory board: Medtronic, Boston Scientific., is co-inventor on a patent application relevant to Deep Brain Stimulation (2014). Speaking fees: Abbvie, Medtronic, Boston Scientific, ECMT. Grant: Stichting Parkinson Fonds. MS: Grants: University Medical Centre Groningen, Stichting Wetenschapsfonds Dystonie Vereniging. JD: Grants: Dutch organizations in scientific research, the Fonds Nutsohra, Jacques and Gloria Gossweiler foundation and Stichting Wetenschapsfonds Dystonie Vereniging. JV: advisory boards: Boston Scientific, Medtronic; grant support: Boston Scientific, Medtronic; Speaking fees: Boston Scientific, Medtronic, St. Jude, UCB, TEVA, and Allergan. MT: Grants: Netherlands organization for scientific research-NWO, Medium, Fonds Nuts-Ohra, Prinses Beatrix Fonds, Gossweiler foundation, Stichting wetenschapsfonds dystonie vereniging, Phelps Stichting, educational grants and national DystonieNet grants from Ipsen and Allergan Famaceutics, Merz, Medtronic and Actelion.
