# Corrigendum: Unmet Needs in the Management of Cervical Dystonia

**DOI:** 10.3389/fneur.2016.00232

**Published:** 2016-12-19

**Authors:** Maria Fiorella Contarino, Marenka Smit, Joost van den Dool, Jens Volkmann, Marina A. J. Tijssen

**Affiliations:** ^1^Department of Neurology, Leiden University Medical Centre, Leiden, Netherlands; ^2^Department of Neurology, Haga Teaching Hospital, Den Haag, Netherlands; ^3^Department of Neurology, University Medical Center Groningen, University of Groningen, Groningen, Netherlands; ^4^Faculty of Health, ACHIEVE Centre of Applied Research, Amsterdam University of Applied Sciences, Amsterdam, Netherlands; ^5^Department of Neurology, Academic Medical Center, Amsterdam, Netherlands; ^6^Department of Neurology, University Clinic of Würzburg, Würzburg, Germany

**Keywords:** cervical dystonia, botulinum toxin, deep brain stimulation, physical therapy modalities, non-motor features

## Description of the Mistake Being Fixed

In the original article, we neglected to thank our sponsor, European Cooperation in Science and Technology (COST), Action BM1101 “European network for the study of dystonia syndromes.” The authors apologize for this oversight.

This error does not change the scientific conclusions of the article in any way.

The correct text of the acknowledgments should read as below.

## Acknowledgments and Funding

This work was supported by European Cooperation in Science and Technology (COST) Action BM1101 “European network for the study of dystonia syndromes”.

## Conflict of Interest Statement

MC, Advisory board: Medtronic, Boston Scientific, is co-inventor on a patent application relevant to Deep Brain Stimulation (2014). Speaking fees: Abbvie, Medtronic, Boston Scientific, ECMT. Grant: Stichting Parkinson Fonds. MS: Grants: University Medical Centre Groningen, Stichting Wetenschapsfonds Dystonie Vereniging. JD: Grants: Dutch organizations in scientific research, the Fonds Nutsohra, Jacques and Gloria Gossweiler foundation and Stichting Wetenschapsfonds Dystonie Vereniging. JV: advisory boards: Boston Scientific, Medtronic; grant support: Boston Scientific, Medtronic; Speaking fees: Boston Scientific, Medtronic, St. Jude, UCB, TEVA, and Allergan. MT: Grants: Netherlands organization for scientific research-NWO, Medium, Fonds NutsOhra, Prinses Beatrix Fonds, Gossweiler foundation, Stichting wetenschapsfonds dystonie vereniging, Phelps Stichting, educational grants and national DystonieNet grants from Ipsen and Allergan Famaceutics, Merz, Medtronic and Actelion.

